# Modeling the Impact of Pandemic Influenza on Pacific Islands

**DOI:** 10.3201/eid1102.040951

**Published:** 2005-02

**Authors:** Nick Wilson, Osman Mansoor, Douglas Lush, Tom Kiedrzynski

**Affiliations:** * Wellington School of Medicine and Health Sciences, Otago University, Wellington, New Zealand;; † Public Health Consulting Ltd, Wellington, New Zealand;; ‡ New Zealand Ministry of Health, Wellington, New Zealand;; § Secretariat of the Pacific Community, Noumea, New Caledonia

**Keywords:** letter, communicable disease control, infection control, influenza, Pacific Islands

**To the Editor:** Many Pacific Island countries and areas have been severely impacted in influenza pandemics. The 1918 pandemic killed substantial proportions of the total population: Fiji ≈5.2%, Tonga ≈4.2% to 8.4%, Guam ≈4.5%, Tahiti ≈10%, and Western Samoa ≈19% to 22% (*1**,**2*). Thirty-one influenza pandemics have occurred since the first pandemic in 1580 (*3*); another one is likely, if not inevitable (*4*). The potential use of influenza as a bioweapon is an additional concern (*5*).

The scale of an influenza pandemic may be projected on the basis of the available historical data that have been built into a computer model, e.g., FluAid (*6*). FluAid uses a deterministic model to estimate the impact range of an influenza pandemic in its first wave. Given the lack of accessible data for specific Pacific Island countries and areas, the default values used in FluAid were used for the proportion of the population in the high-risk category for each age group, for the death rates, hospitalizations, and illness requiring medical consultations. Country-specific population data were obtained from the Secretariat of the Pacific Community, and hospital bed data were obtained from the World Health Organization (WHO) (*7**,**8*). The FluAid model was supplemented by a model of an 8-week pandemic wave and modeling of hospital bed capacity. Further methodologic details are provided in the Appendix.

The results indicate that at incidence rates of 15% and 35%, pandemic influenza would cause 650 and 1,530 deaths, respectively, giving crude death rates of 22 to 52 per 100,000 (Table A1). Most deaths (83%) would occur in the high-risk group, 60% of whom would be 19–64 years of age, and 22% would be >65 years of age. Additionally, 3,540 to 8,250 persons would be hospitalized, most of whom (78%) would not have high-risk conditions. Also, 241,000 to 563,000 medical consultations would occur. Most (87%) consultations would be for patients without high-risk conditions (50% birth–18 years of age and 46% 19–64 years of age).

In the peak week of the pandemic (week 4), from 15% to 34% of all hospital beds would be required for patients with influenza (Table). The upper end of impact on hospital beds at >40% would occur for Guam, Kiribati, Marshall Islands, Northern Mariana Islands, and Tonga. Assuming all consultations required doctors, 42 to 99 influenza consultations per doctor would be required during the peak week (Table). The upper end of impact on consultations for individual Pacific Island countries and areas would vary from 31 (New Caledonia) to 524 (Vanuatu); Fiji, Kiribati, Samoa, Solomon Islands, Tonga, and Vanuatu would have rates >150 consultations per week.

The uncertainties associated with pandemic influenza mean that any modeling of its future impact is relatively crude. For example, the new strain may be particularly infectious, virulent, or both. In contrast, the use of international-level public health interventions as recommended by WHO (*9*) may prevent pandemic influenza from reaching some Pacific Island countries and areas or particularly remote island groups. These issues and other limitations with the model are detailed in the Appendix.

Nevertheless, if the death rate is in the range suggested by the model, this outcome would make it the worst internal demographic event since the 1918 influenza pandemic for many Pacific Island countries and areas. The lower death rate (albeit for a single wave) is similar to the U.S. rates for the 1957 influenza pandemic (22 per 100,000) and the 1968 influenza pandemic (14 per 100,000) (*10*). The upper end is considerably lower than for the 1918 pandemic, which suggests that the range indicated is reasonably plausible. Although relatively high, the death toll from pandemic influenza would still be less than the typical annual impact for some Pacific Island countries and areas from other infectious diseases (including malaria and diarrheal diseases) and from such fundamental determinants of health status such as poor sanitation, poor diet, and tobacco use.

The predicted range of hospitalizations attributable to pandemic influenza would likely overwhelm hospital capacity in many of the Pacific Island countries and areas. Rapid response at the onset of the pandemic could ensure efficacious use of hospital beds and resources, e.g., cancel elective procedures and early discharge to community care. Other contingency plans by hospitals could facilitate lower hospital admission rates (e.g., strengthening the primary care response).

Planning and capacity building could be provided by WHO, the Secretariat of the Pacific Community, and donor nations and agencies with support for improving surveillance and other preventive measures for disease control (see Appendix for details). A combination of national capacity building with international support will maximize the capacity to respond to the next influenza pandemic as well as other potential communicable disease threats.

## Appendix

### Additional Methods

The output of the FluAid model is the number of additional deaths, hospitalizations, and illness attributable to pandemic influenza that will require medical consultations. The model assumes no effective public health interventions to control disease spread (such as quarantine, access to appropriate vaccine, or widespread use of antiviral drugs). Specific details about the FluAid software and the various assumptions in the model are detailed on the Centers for Disease Control and Prevention (CDC) Web site (*11*) and in other documents (*12**,**13*).

Given the lack of accessible data for specific Pacific Island countries and areas, the default values used in FluAid were used to determine the proportion of the population in the high-risk category for each age group, when calculating the death rates, the hospitalization rates, and the illness rates. The model's parameters were based on the available data (mostly from North America and some from Europe) from the 1957 pandemic and subsequent nonpandemic data (*12*). Persons categorized as high-risk have a preexisting medical condition (e.g., diabetes) that makes them more susceptible to developing medical complications due to influenza. The proportions in this high-risk category used in the model were 6.4% for persons birth–18 years of age, 14.4% for those 19–64 years of age, and 40% for those ≥65 years of age. The output values from the model were for most likely, minimum, and maximum values (each for death, hospitalization, and illness requiring medical consultations).

The FluAid model provides the total disease impact but does not specify its time distribution. The length of influenza epidemics is highly variable (*14**,**15*), but for this analysis, the first pandemic wave was assumed to span 8 weeks and have the same distribution over time as a model of a stochastically simulated influenza epidemic (*16*) i.e., 32.3% of all cases in week 4, the peak week.

The countries and areas included in this analysis were the 20 tropical Pacific Island countries and areas that are members of the Secretariat of the Pacific Community (SPC) (excluding Papua New Guinea, and Pitcairn Island). Mid-2004 total population estimates from SPC (*17*) were used and adjusted according to World Health Organization (WHO) data on population distribution (*18*) to fit the age categories required by the model.

The total number of hospital beds in each Pacific Island country and area was based on WHO data (*19*). However, the data for Tonga were updated from more recent information (S. Kupu, pers. comm.) and updated similarly for Niue as well (*20*). When countries' data were missing, the information was found through additional Internet searches (i.e., for Nauru and Tokelau). The number of doctors per capita was also obtained from WHO data (*19*).

### Limitations with the Modeling

The uncertainties associated with pandemic influenza mean that any modeling of its future impact is relatively crude. The FluAid model also has a number of specific limitations, which may lend an underestimation of the next pandemic's impact. First, the new strain may be particularly infectious, virulent, or both. The model's upper incidence rate for clinical illness was 35%, when higher rates (e.g., 50%) are plausible. Second, the proportions of the population in various high-risk groups in Pacific Island countries and areas may be larger than used in the model. This is plausible given the relatively high prevalence of chronic conditions such as diabetes in some Pacific Island countries and areas (e.g., a 23% prevalence rate for diabetes amongst adults aged 25–64 years in Samoa [21]). Finally, the level of antimicrobial drug resistance (e.g., *Streptococcus pneumoniae*) may continue to increase globally, thus the actual death rate may be higher than used in this model (i.e., if alternative treatments for the secondary microbial infections after influenza infection are not readily available).

In contrast, the results could also be overestimated for the following reasons: First, the use of international level public health interventions as recommended by WHO (*22*) may prevent or delay pandemic influenza reaching some Pacific Island countries and areas or particularly remote island groups (e.g., the provision of health alert notices to incoming travelers and entry screening). Improvements in surveillance systems (with access to rapid detection kits) over time may increase the chances of control measures being successful or subsequent pandemic waves being delayed. Second, some Pacific Island countries and areas could possibly avoid the first pandemic wave and might have access to a vaccine for protection from subsequent pandemic waves (though this may take 6–9 months from the time that a new virus variant is first identified (*23*). In addition, the use of antiviral agents (*24*) could prevent infection and reduce illness among key personnel and also those with high-risk conditions, but only if supplies are adequate. A recent study suggests that pandemic influenza could be contained with "the use of antiviral prophylaxis, if 80% of the exposed persons maintained prophylaxis for up to 8 weeks" (*16*).

Also, improved treatment in the community and hospital settings could lower hospitalization and death rates (relative to those used in this model). Finally, the geographic dispersal of some Pacific Island countries and areas may mean that spread within the country is much slower than the 8 weeks used in this model. This would reduce the peak demand on health services.

### Possible Roles of WHO, SPC, and Donor Nations and Agencies

Agencies such as WHO and SPC play valuable roles in improving influenza surveillance, which may facilitate the control of pandemic influenza. Donor nations and agencies can potentially contribute to enhancing regional surveillance efforts by sharing their experience with developing national influenza pandemic plans and by conducting pandemic planning exercises (New Zealand has experience with both of these [25,26]).

Donor support for increasing the size of the health workforce could assist not only at a time of a pandemic but also when dealing with the current threats to health. Such support could include additional funds for health workforce training and placing and retaining staff in areas of greatest need (e.g., remote locations). Reducing the burden of chronic disease (e.g., donor support for enhanced tobacco control) may also reduce the proportion of the population at increased risk of adverse sequelae from infection with influenza.

Investment by donors in expanding current hospital bed capacity in Pacific Island countries and areas may be of value. However, this is likely a lower priority than strengthening primary healthcare services in Pacific Island countries and areas and making plans to mobilize effective community care. Funding for antiviral drugs and influenza vaccine (when available) to healthcare workers and high-risk groups could also be considered.

## Appendix

**Table A1:** Deaths, hospitalizations, and medical consultations predicted for the next influenza pandemic using the FluAid model (at incidence rates [IR] of clinical illness of 15% and 35%)

Country/area	Deaths (15% IR)		Deaths (35% IR)		Hospitalizations (15% IR)		Hospitalizations (35% IR)		Consultations (15% IR)		Consultations (35% IR)	
	Most likely	Range	Most likely	Range	Most likely	Range	Most likely	Range	Most likely	Range	Most likely	Range
Melanesia												
Fiji Islands	195	68–402	453	160–937	1,063	293–1,490	2,480	684–3,476	68,604	53,442–93,423	160,077	124,698 – 217,988
New Caledonia	64	28–124	152	64–292	332	99–449	773	232–1,048	19,311	15,019–26,652	45,060	35,044 – 62,186
Solomon Islands	82	33–190	192	79–444	459	143–749	1,071	332–1,750	38,667	30,869–50,274	90,223	72,028 – 117,308
Vanuatu	42	16–94	99	38–219	235	70–363	549	162–846	17,988	14,241–23,765	41,973	33,230 – 55,452
Micronesia												
Federated States of Micronesia	24	9–50	56	21–119	129	39–193	300	89–452	9,358	7,384–12,458	21,834	17,231 – 29,069
Guam	43	21–85	101	48–197	218	69–308	507	163–719	13,670	10,754–18,527	31,895	25,092 – 43,230
Kiribati	19	7–42	44	17–97	104	31–158	243	72–369	7,746	6125–10,271	18,074	14,290 – 23,966
Marshall Islands	10	3–22	23	8–54	58	16–90	137	38–211	4,621	3,650– 6,096	10,782	8,516 – 14,223
Nauru	1	0–4	4	1–10	11	3–16	25	7–38	842	665–1,111	1,965	1,549 – 2,593
Northern Mariana Islands	18	4–39	44	11–89	109	25–141	253	59–329	6,304	4,807–8,833	14,711	11,215 – 20,609
Palau	6	3–11	13	5–26	30	9–40	70	20–92	1,683	1,305–2,335	3,926	3,044 – 5,448
Polynesia												
American Samoa	12	4–28	31	12–67	73	20–107	169	49–251	5,186	4,080–6,933	12,101	9,521 – 16,178
Cook Islands	5	2–8	10	5–19	20	7–27	47	15–65	1,139	887–1,579	2,657	2,069 – 3,685
French Polynesia	59	19–121	137	42–281	327	84–447	763	197–1,042	20,461	15,827–28,107	47,742	36,931 – 65,583
Niue	1	0–1	1	1–2	2	1–3	5	2–7	131	103–179	306	241 – 419
Samoa	40	19–84	95	42–198	212	67–319	495	157–746	15,186	12,034–20,182	35,435	28,078 – 47,091
Tokelau	1	0–1	1	1–2	2	1–3	4	2–6	125	100–165	292	233 – 386
Tonga	25	11–49	57	27–113	124	39–180	290	93–418	8,115	6,402–10931	18,937	14,936 – 25,506
Tuvalu	3	1–5	6	3–12	13	5–18	30	10–42	788	619–1,074	1,838	1,445 – 2,505
Wallis and Futuna	3	1–8	8	4–18	18	5–26	44	14–62	1,231	971–1,656	2,873	2,265 – 3,862
Total	653	252–1369	1,527	588–3,195	3,539	1,027–5,130	8,255	2,395–11,971	241,156	189,283–324,553	562,701	441,660 – 757,289

**Table:** Predicted impact on health services from the next influenza pandemic using the FluAid model for the peak week (at incidence rates [IR] of 15% and 35%)

Country/area	Input data		Hospital bed requirement in the peak week (% of bed capacity)		Consultations per physician in the peak week	
	Hospital beds (n)	Physicians per 10,000 population	15% IR	35% IR	15% IR	35% IR
Melanesia						
Fiji Islands	2,097	3.4	16	38	78	182
New Caledonia	935	20.1	11	27	13	31
Solomon Islands	881	1.3	17	39	209	487
Vanuatu	605	1.2	13	29	224	524
Micronesia						
Federated States of Micronesia	329	5.9	13	29	45	106
Guam	225	11.1	31	73	24	56
Kiribati	140	3.0	24	56	90	209
Marshall Islands	105	4.6	18	42	59	137
Nauru	50	15.7	7	16	17	40
Northern Mariana Islands	82	4.5	43	100	58	135
Palau	90	11.0	11	25	24	56
Polynesia						
American Samoa	140	7.0	17	39	38	89
Cook Islands	128	7.8	5	12	34	79
French Polynesia	1,062	17.5	10	23	15	35
Niue	0	13.0	-*	-*	20	48
Samoa	557	3.4	12	29	79	184
Tokelau	36	13.3	2	4	20	47
Tonga	200	3.5	20	47	76	178
Tuvalu	56	5.9	7	17	45	105
Wallis and Futuna	75	9.2	8	19	29	68
Total	7,793	6.3	15	34	42	99

*The single hospital in Niue was completely destroyed in a cyclone in 2004.
